# Identification of Genes that Elicit Disuse Muscle Atrophy via the Transcription Factors p50 and Bcl-3

**DOI:** 10.1371/journal.pone.0016171

**Published:** 2011-01-13

**Authors:** Chia-Ling Wu, Susan C. Kandarian, Robert W. Jackman

**Affiliations:** Department of Health Sciences, Boston University, Boston, Massachusetts, United States of America; University Hospital Vall d'Hebron, Spain

## Abstract

Skeletal muscle atrophy is a debilitating condition associated with weakness, fatigue, and reduced functional capacity. Nuclear factor-kappaB (NF-κB) transcription factors play a critical role in atrophy. Knockout of genes encoding p50 or the NF-κB co-transactivator, Bcl-3, abolish disuse atrophy and thus they are NF-κB factors required for disuse atrophy. We do not know however, the genes targeted by NF-κB that produce the atrophied phenotype. Here we identify the genes required to produce disuse atrophy using gene expression profiling in wild type compared to *Nfkb1* (gene encodes p50) and *Bcl-3* deficient mice. There were 185 and 240 genes upregulated in wild type mice due to unloading, that were not upregulated in *Nfkb1^−/−^* and *Bcl-3^−/−^* mice, respectively, and so these genes were considered direct or indirect targets of p50 and Bcl-3. All of the p50 gene targets were contained in the Bcl-3 gene target list. Most genes were involved with protein degradation, signaling, translation, transcription, and transport. To identify direct targets of p50 and Bcl-3 we performed chromatin immunoprecipitation of selected genes previously shown to have roles in atrophy. *Trim63 (MuRF1)*, *Fbxo32* (*MAFbx*), *Ubc*, *Ctsl*, *Runx1*, *Tnfrsf12a* (Tweak receptor), and Cxcl10 (*IP-10*) showed increased Bcl-3 binding to κB sites in unloaded muscle and thus were direct targets of Bcl-3. p50 binding to the same sites on these genes either did not change or increased, supporting the idea of p50:Bcl-3 binding complexes. p65 binding to κB sites showed decreased or no binding to these genes with unloading. *Fbxo9*, *Psma6*, *Psmc4*, *Psmg4*, *Foxo3*, *Ankrd1* (CARP), and *Eif4ebp1* did not show changes in p65, p50, or Bcl-3 binding to κB sites, and so were considered indirect targets of p50 and Bcl-3. This work represents the first study to use a global approach to identify genes required to produce the atrophied phenotype with disuse.

## Introduction

Skeletal muscle atrophy is a highly regulated process in which the size of a multinucleated fiber is controlled by signaling that regulates gene expression from triggers in the muscle [1], [2]. One group of signaling proteins that regulate muscle atrophy is the nuclear factor-kappaB (NF-κB) family of transcription factors. A role for NF-κB in adult muscle atrophy or wasting has been found in aging [3], [4], disuse [5], [6], [7], [8], denervation [9], [10], muscular dystrophy [11], [12], and cachexia due to illnesses such as cancer [10], [13], [14]. NF-κB transcription factors (p65, c-Rel, RelB, p52, p50) act as dimers that bind target genes that have been shown to regulate cellular processes as diverse as immunity, inflammation, development, cell proliferation, and apoptosis [15], [16], [17].

In the case of disuse atrophy, NF-κB is necessary for muscle fiber wasting. Evidence for this comes from data showing that a NF-κB reporter is strongly activated in muscle fibers at 3, 7, and 10 days of disuse by hind limb unloading [5], [7], [8]. Inhibition of components of upstream NF-κB regulatory proteins such as the overexpression of a super repressor form of the inhibitor of κB (IκBα SR) showed a 40% inhibition of fiber atrophy [5], and overexpression of either a dominant negative (d.n.) form of IκB kinase beta (IKKβ) or a d.n. form of IKKα each showed a 50% inhibition of fiber atrophy and a complete abolition of unloading induced NF-κB reporter [6]. Importantly, a 70–100% inhibition of muscle fiber atrophy was found in mice lacking either the *Nfkb1* gene (encodes the NF-κB transcription factor, p50) or the *Bcl-3* gene, which encodes a NF-κB co-transactivator [7]. Gel supershift assays and nuclear levels of p65, p52, and RelB do not suggest significant involvement of these NF-κB family members [8]. Rel knockout mice showed that c-Rel is not required for disuse atrophy [5]. An important feature of NF-κB activation in disuse-induced atrophy is that there is no evidence of inflammation or complement activation [8], [18], [19] as is seen with other types of muscle atrophy such as that due to primary muscle disease, systemic illness, or aging [20]. When inflammation is associated with muscle wasting, involvement of the prototypical NF-κB family member p65 (Rel A) has been evident [3], [4], [10], [11], [12], [13], [20].

Although we have found that upstream signaling proteins, such as IκBα, IKKα and IKKβ that activate NF-κB transcription are required for atrophy, we do not understand how NF-κB transcription factors produce the atrophied phenotype. We do know that the transcription factors p50 and Bcl-3 are essential for atrophy during disuse [7], so in the present study we first identified the genes being targeted by p50 and Bcl-3, thereby identifying genes that are required to produce the atrophied phenotype in each case. To do this we performed global gene expression analysis of plantar flexor muscles from weight bearing and hind limb unloaded wild type mice and compared these data to the same muscles from *Nfkb1* ^−/−^ mice and from *Bcl3^−/−^* mice. This provided a list of genes required for atrophy, and that are considered either direct or indirect targets of p50 and Bcl-3 during atrophy. From the lists we selected genes to study in further detail to determine whether p50 and/or Bcl-3 showed increased binding to κB binding sites using chromatin immunoprecipitation (ChIP) assays in weight bearing and unloaded mouse plantar flexor muscles. An advantage of using gene expression profiling in combination with ChIP assays is that they reflect mRNA expression and DNA binding in vivo, at the moment of isolation. A gene showing increased mRNA expression in wild type muscle but not in knockout muscle due to unloading *and* that shows an increase in p50 and/or Bcl-3 binding in wild type unloaded muscle is evidence that a p50 or Bcl-3 direct target gene has been identified.

This work represents the first study to use a global approach to identify the genes required to produce the atrophied phenotype due to muscle unloading. Identification of genes that are NF-κB targets is a first step in the discovery of how these transcription factors produce the atrophied phenotype during skeletal muscle disuse.

## Results

### Muscle Atrophy

There was an 18% decrease in gastrocnemius plus plantaris muscle mass for wild type 6-day hind limb unloaded mice (wet weight normalized to body weight), but there were little to no differences for the same muscles in the *Nfkb1^−/−^* or *Bcl-3^−/−^* mice (Table S1). This is consistent with the atrophy we reported previously in plantaris muscles due to 10 days of unloading in wild type mice, but little to no differences in plantaris muscle atrophy in the *Nfkb1^−/−^* or *Bcl3^−/−^* mice [7].

### Microarray analysis of muscle from wild type and knockout mice to identify direct or indirect targets of p50 and Bcl-3 in atrophy

A major aim of this study was to identify genes from the atrophied phenotype with unloading. Since *Nfkb1^−/−^* or *Bcl-3^−/−^* mice showed little or no atrophy with unloading [7], finding the genes that drive the atrophy process was accomplished by identifying those genes that were upregulated in the unloaded muscles from wild type mice but not upregulated in the unloaded muscles from *Nfkb1^−/−^* or *Bcl-3^−/−^* mice. The reason for focusing on upregulated genes is that previous work showed a marked upregulation of NF-κB dependent binding and transcription in atrophying muscles [7], [8]. The expression data for up and down-regulated genes due to hind limb unloading for all 3 strains of mice (wild type, *Nfkb1^−/−^*, *Bcl3^−/−^*) have been deposited in the MIAME compliant Gene Expression Omnibus (http://www.ncbi.nlm.nih.gov/geo/), accession number GSE23497.

Using the statistical methods described below, we identified upregulated genes in 6-day unloaded muscle in wild type but not in knockout mice. There were 185 genes upregulated in wild type mice but not in *Nfkb1^−/−^* mice due to unloading and these genes were thus considered either direct or indirect targets of the p50 transcription factor during muscle atrophy (Table S2). Functional classification of these genes was made based on the molecular function and biological processes annotated by the Gene Ontology Consortium (Table 1). Most genes were involved in signaling (15%), translation (10%), metabolism (10%), protein degradation (9%), transcription (8%), and transport (6%). The same approach was used to identify direct or indirect targets of Bcl-3 using *Bcl3^−/−^* mice. This produced a list of 240 genes that were direct or indirect targets (Table S2) of this transcriptional co-activator that is known to bind p50 dimers to induce transcription [21], [22], [23]. These genes also belong largely to signaling (13%), metabolism (10%), translation (9%), protein degradation (9%), transcription (8%), and transport (8%) (Table 1). Upregulation of genes in these functional categories is consistent with the changes seen in other studies using microarray data to describe muscle atrophy [18], [24].

**Table 1:** Functional category of target genes for Bcl-3 and p50 from microarray analysis.

	Bcl3 targets		p50 targets	
	Number	Percentage	Number	Percentage
Signaling	31	12.92%	28	15.14%
Metabolism	24	10.00%	18	9.73%
Translation	22	9.17%	19	10.27%
Protein degradation	21	8.75%	17	9.19%
Transcription	21	8.75%	16	8.65%
Transport	19	7.92%	12	6.49%
Non-annotated or RIKEN cDNA	14	5.83%	9	4.86%
Misc: immune/proliferation/ECM	13	5.42%	11	5.95%
RNA processing	12	5.00%	9	4.86%
Cytoskeleton	11	4.58%	9	4.86%
Chromatin modification	10	4.17%	7	3.78%
Cell adhesion	7	2.92%	6	3.24%
Structure	7	2.92%	5	2.70%
Development	7	2.92%	4	2.16%
Cell cycle	6	2.50%	4	2.16%
Binding	5	2.08%	4	2.16%
Anti-oxidant	4	1.67%	3	1.62%
Muscle contraction	3	1.25%	3	1.62%
Stress	3	1.25%	1	0.54%

### Selected Genes for Further Study as p50/Bcl-3 Targets

From the microarray data, we identified 25 upregulated genes that were targets of p50 and/or Bcl-3 and that have at least some known or inferred function in muscle atrophy (Table 2). All but 6 of these 25 genes are involved in some aspect of protein degradation. Seven are involved in ubiquitination of protein substrates (*Fbxo32*, *Fbxo9*, *Fbxo36*, *Ubc*, *Ube2j1*, *Nedd4l*, *Cul4a*), 9 encode proteasomal subunits (*Psma7*, *Psmb3*, *Psmc4*, *Psmd2*, *Psmd13*, *Psmb4*, *Psmd4*, *Psmg4*, *Psma6*), 2 are lysosomal genes (*Ctsl*, *Ctss*), and one is involved in calpain mediated protein cleavage (*Capns1*). There were 4 genes whose protein product can act as a transcription factor (*Ankrd1* (*Carp*), *Runx1*, *Jun*, *Foxo3*), a gene encoding a translational repressor (*Eif4ebp1*), and the Tweak cytokine receptor (*Tnfrsf12a*). Twelve of these 25 genes, a representative sampling of the functional groups, plus 2 other genes that were not on the microarray, *Trim63* (*MuRF1*) and *Cxcl10* (*IP-10*), were chosen for study in more detail (14 genes total).

**Table 2:** Selected genes with known or inferred functions in muscle atrophy that were target genes for Bcl-3 and/or p50.

Probe ID	Gene Symbol	FC in WT	Bcl-3 Target	p50 Target
**Ubiquitination**				
1417522_at	Fbxo32	2.9	Y	Y
1432211_a_at	Fbxo9	1.8	Y	N
1419305_a_at	Fbxo36	2.4	Y	Y
1425966_x_at	Ubc*	1.8	Y	Y
1448824_at	Ube2j1	1.6	Y	Y
1423269_a_at	Nedd4l	1.5	Y	Y
1451971_at	Cul4a	1.5	Y	Y
**Proteasomal subunits**				
1423568_at	Psma7	1.5	Y	Y
1460198_a_at	Psmb3	1.5	Y	Y
1416290_a_at	Psmc4	1.5	Y	Y
1415831_at	Psmd2	1.5	Y	Y
1422459_a_at	Psmd13	1.5	Y	Y
1438984_x_at	Psmb4	1.4	Y	Y
1425859_a_at	Psmd4	1.4	Y	Y
1435431_at	Psmg4	1.4	Y	Y
1435317_x_at	Psma6	2.3	Y	Y
**Lysosomal proteins**				
1451310_a_at	Ctsl	1.6	Y	Y
1448591_at	Ctss	1.5	Y	Y
**Transcription factors**				
1420992_at	Ankrd1	7.9	Y	Y
1422864_at	Runx1	5.3	Y	Y
1417409_at	Jun	1.7	Y	Y
1434832_at	Foxo3*	1.6	Y	Y
**Others**				
1418572_x_at	Tnfrsf12a	2.7	Y	Y
1434976_x_at	Eif4ebp1	1.6	Y	Y
1426400_a_at	Capns1	1.5	Y	Y

FC="fold change” of gene expression in weight bearing vs. hind limb unloaded groups in wild type animals from microarray analysis. Y = a target gene for Bcl-3 or p50, N = not a target gene for Bcl-3 or p50.

*gene significantly increased by t-test but did not pass multiple test correction.

### Quantitative real-time PCR of selected atrophy genes

**Figure 1:**
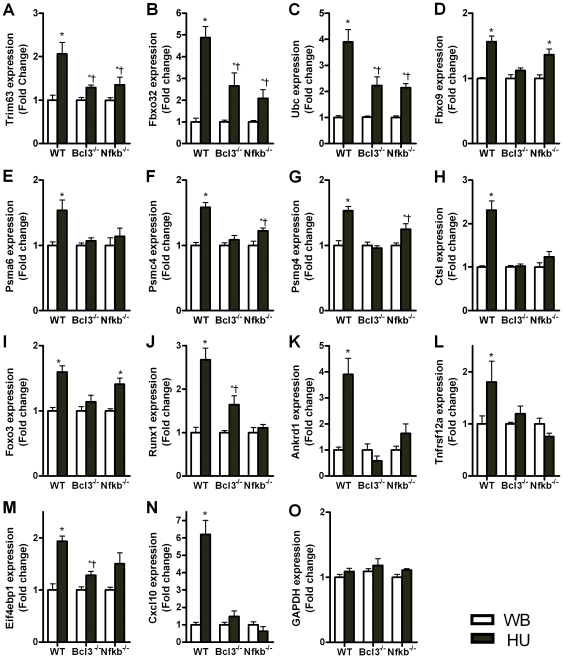
Quantitative real time PCR (qPCR) to measure mRNA expression of selected atrophy genes. Taqman probe and primer sets were used to confirm mRNA expression from microarray data of gastrocnemius and plantaris muscles. All 14 genes measured confirmed a significant upregulation due to 6 days unloading (HU) compared to weight bearing (WB) in muscles of wild type (WT) mice. Gene expression due to unloading in knockout mice (*Nfkb1* and *Bcl-3*) was either not different from weight bearing or it had a significantly less increase for all cases except Fbxo9 and Foxo3 in *Nfkb1^−/−^* mice. Gene expression was measured for: **A**) Trim63 (MuRF1), **B**) Fbxo32 (MAFbx), **C**) ubiquitin C, **D**) Fbxo9, **E**) Psma6, **F**) Psmc4, **G**) Psmg4, **H**) Ctsl, **I**) Foxo3, **J**) Runx1, **K**) Ankrd1 (Carp), **L**) Tnfrsf12a (Tweak receptor), **M**) Eif4ebp1, **N**) Cxcl10 (IP-10), and **O**) GAPDH. *significantly different from weight bearing value (*P*<0.05), †significantly different from wild type unloaded value (*P*<0.05).

Quantitative real-time PCR (qPCR) confirmed significant upregulation by unloading of the 14 genes selected for further study and their lack of upregulation in knockout mice (Figure 1). Many of these genes are involved in ubiquitin-proteasome mediated protein degradation, namely, *Trim63 (MuRF1)*, *Fbxo32 (MAFbx)*, *Ubc*, *Fbxo 9*, *Psma6*, *Psmc4*, and *Psmg4* (Figure 1A–G). Other genes whose microarray data were confirmed by qPCR and that play a role in muscle atrophy were *Ctsl*, *Foxo3*, *Runx1*, *Ankrd1*, *Tnfrsf12a*, and *Eif4ebp1* (Figure 1H–M). The *Trim63* gene was not on our microarray but has a significant role in muscle atrophy [25], [26], [27], [28]. Another gene not on the microarray, *Cxcl10* (*IP-10*), was also studied (Figure 1N) because it was found to be strongly upregulated in unpublished work and in response to TNFα; it is a known p50:Bcl-3 target in immune cells [29]. Almost all genes were shown to be direct or indirect targets of Bcl-3 and p50 since there was either no upregulation, or significantly less upregulation, in unloaded muscles from *Bcl3^−/−^* and *Nfkb1^−/−^* mice compared to unloaded muscles from wild type mice. The exceptions were Fbxo9 and Foxo3, which showed upregulation in unloaded muscle of *Nfkb1^−/−^* mice similar to that of wild type mice (Figure 1D, I), so these two genes were not confirmed targets of p50 during unloading. GAPDH did not change with unloading in wild type or mutant mice and so it was used for normalization of all the mRNAs measured by qPCR (Figure 1O).

### Chromatin Immunoprecipitation (ChIP) of selected p50 or Bcl-3 putative target genes

For genes that were direct or indirect targets of p50 and Bcl-3 with muscle unloading, we performed ChIP assays using muscle from wild type mice to assess whether there was a commensurate change in p50 and Bcl-3 binding to NF-κB binding sites due to muscle unloading. For comparison, we also measured changes in the prototypical NF-κB transcription factor family member, p65, because there are not definitive data as to whether it is involved in NF-κB dependent gene regulation by muscle unloading as there is with Bcl-3 and p50. To identify putative κB binding sites in each gene to be studied, an algorithm called CLOVER [30] was used to search for sites in the genomic sequence from 10kb upstream of each gene's transcription start site (TSS) to 10kb downstream from its polyadenylation signal.

**Figure 2:**
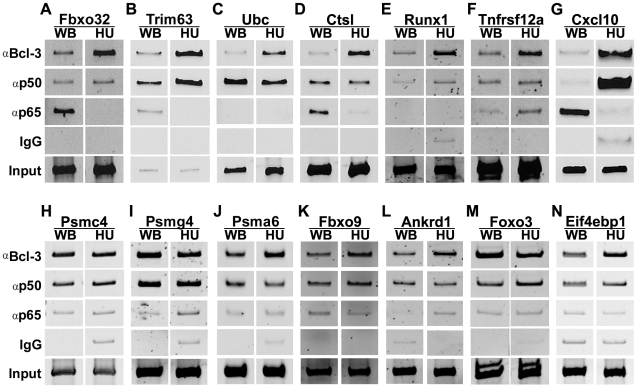
ChIP assays of Bcl-3, p50, and p65 protein binding to genes from **Figure 1**. ChIP assay was performed using gastrocnemius/plantaris muscle from weight bearing (WB) and unloaded (HU) mice. For each gene shown, binding of Bcl-3, p50 and p65 protein to a κB binding site was measured. ChIP showed an increase in Bcl-3 protein binding to 7 of the 14 genes studied, these were: **A**) *Fbxo32*, **B**) *Trim63*, **C**) *Ubc*, **D**) *Ctsl*, **E**) *Runx1*, **F**) *Tnfrsf12a*, and **G**) *Cxcl10*. There were also increases in p50 (**B, E, G**) and decreases in p65 (**A, B, D, G**) in these 7 genes. None of the proteasomal subunit genes showed changes in Bcl-3, p50, or p65 binding, *Psmc4*., *Psmg4*, or *Psma6* (**H–J**). *Fbxo9*, *Ankrd1*, *Foxo3*, and *Eif4ebp1* (**K–N**) genes also did not show changes in the binding of these proteins to κB sites. For each gene, ChIP assays were repeated using different samples from WB and HU groups. The PCR product size for all 14 ChIP assays performed was between 190 to 400bp.

Of the 14 genes on which a ChIP assay was performed, 11 had conserved κB sites. The other 3 genes contained κB sites, but none were conserved (*Fbxo9*, *Psmg4*, *Eif4ebp1*); not surprisingly, these 3 genes did not show changes in binding of the 3 κB factors studied, due to unloading. The most frequent and robust change in protein binding to κB sites of the 3 transcription factors studied was Bcl-3. There was an increase in Bcl-3 binding to conserved κB sites in 7 of the 14 genes studied. These were *Fbxo32* (the site 550bp upstream of the TSS), *Trim63* (the site in the third intron 4.8 kb downstream of the TSS), *Ubc* (the site 1.4 kb upstream of the TSS), *Ctsl* (the site 5.5 kb upstream of the TSS), *Runx1* (the site 1.7 kb upstream of the TSS), *Tnfrsf12a* (the site 5.7 kb upstream of the TSS), and *Cxcl10* (the two sites 157 bp upstream of the TSS) (Figure 2A–G). Of these 7 genes, p50 binding to the same κB site was either unchanged or, it was increased in *Trim63*, *Runx1*, and *Cxcl10*. In all 7 genes, the increased Bcl-3 binding to sites where p50 was bound suggests increased formation of p50-Bcl-3 complexes with unloading. For p65 binding there were marked decreases in 4 genes, *Fbxo32*, *Trim63*, *Ctsl*, and *Cxcl10* (Figure 2A, B, D, G) and no detectable binding in control or unloaded muscle in *Ubc* or *Runx1* (Figure 2C, E). The fact that no κB site showed marked p65 binding with unloading supports earlier work suggesting that p65 is not a major player in disuse muscle atrophy [8]. There was a moderate increase in p65 binding in one gene, *Tnfrsf12a* (Figure 2F). Each immunoprecipitation was repeated with different muscles from weight bearing and unloaded groups.

The proteasome subunit genes studied, *Psmc4*, *Psmg4*, and *Psma6* (Figure 2H–J), and located 1.9, 2.7, and 3.2kb upstream from the TSS, respectively, were not direct κB target genes since p65, p50 or Bcl-3 showed no change in binding. The κB sites studied in *Fbxo9*, *Ankrd1*, *Foxo3*, *and Eif4ebp1* (Figure 2K–N) genes, located at 1.2, 2.3, 2.0, and 2.7kb upstream of the TSS, respectively, also did not show significant changes in binding of Bcl-3, p50, or p65. None of these 7 genes appear to be direct p50 or Bcl-3 target genes.

## Discussion

This study is the first to identify on a global scale, direct or indirect target genes of two transcription factors previously shown to be required for muscle disuse atrophy, p50 and Bcl-3 [7]. This was done using gene expression profiling of unloaded skeletal muscle from wild type mice compared to unloaded muscle from mice deficient for the gene encoding each of these transcription factors. Genes in wild type mice upregulated by hind limb unloading that are not upregulated in unloading of knockout mice are either direct or indirect targets of the gene knocked out. Since our previous work shows a complete atrophy inhibition when the genes encoding p50 and Bcl-3 are knocked out, it is not surprising to have a significant number (∼200) of genes that are targets of these transcription factors. In combination with these data, we performed ChIP assays on κB sites in 14 of the κB -upregulated genes (verified by qPCR) to assess whether they were direct targets of p50 and Bcl-3. We focused on upregulated genes because disuse elicits an increase in NF-κB binding to DNA and a robust increase in NF-κB dependent transcription in unloaded muscle from rats and mice [5], [6], [7], [8].

The microarray and ChIP results show that *Fbxo32*, *Trim63*, *Ubc*, *Ctsl*, *Runx1*, *Tnfrsf12a*, and *Cxcl10* are direct gene targets of Bcl-3 during disuse atrophy because of their lack of mRNA upregulation in *Bcl-3^−/−^* mice with unloading and the increase in Bcl-3 binding to conserved κB sites in unloaded muscles of wild type mice. Since Bcl-3 does not directly bind DNA, we presume that it bound to existing p50 binding at the κB site under study. All genes studied showed p50 binding in control muscle, and with unloading, p50 binding either did not change or increased in the 7 genes where Bcl-3 binding was increased. We interpret these data to indicate the formation of p50:Bcl-3 complexes. Other data supporting the idea of p50 and Bcl-3 working together to effect gene expression changes with unloading is that all of the p50 targets revealed by the microarray data were also Bcl-3 target genes. Moreover, the increase in NF-κB reporter activity due to unloading in wild type mice is absent in unloaded muscle from *Nfkb1^−/−^* and *Bcl-3^−/−^* mice [7]. In unloaded muscle, binding of the prototypical κB protein p65 decreased markedly or was not detectable in all but one of these 7 genes. Taken together, these data suggest p50:Bcl-3 complexes are important proteins binding to κB sites during muscle atrophy. There is a significant literature on the ability of p50 homodimers to induce transactivation when bound to Bcl-3 in several non-muscle cell types [21], [22], [23].

There was a marked increase in Bcl-3 binding to conserved κB sites in 4 upregulated genes with known roles in protein degradation with muscle atrophy. Thus, they are direct targets of κB. These are *Fbxo32* [28], [31] *Trim63* [25], , *Ubc* [18], [24], [28], [32], and *Ctsl* [5], [24], [33]. Although we now clearly show that p50 and Bcl-3 target these genes and that NF-κB is required for their upregulation, there may be other factors involved. For instance, FoxO3 was shown to target *Fbxo32* during starvation atrophy [34]. FoxO1 has been shown to target *Ctsl* during starvation atrophy and regulate its transcription [33]. NF-κB was shown to target *Trim63* in skeletal muscle in cachexia [10] and the present work supports these data in atrophying muscle due to unloading, although we show a role for p50 and Bcl-3 rather than involvement of p65.

It was interesting however, that three of the nine proteasomal genes shown to be targets of p50 and Bcl-3, and chosen for further study, were indirect rather than direct κB target genes because there was no change in p65, p50, or Bcl-3 binding with unloading. This supports the idea that proteasomal subunit upregulation with atrophy is directly regulated by transcription factors other than NF-κB, although NF-κB may be regulating these unknown factors based on the gene expression data in the knockout mice.

Another direct target gene of NF-κB proteins is *Runx1*, previously shown to moderate myofibrillar stabilization and autophagy during disuse atrophy [35]. It is a transcription factor with 29 putative gene targets in atrophying muscle. These targets appear to be mostly structural genes, distinct from the structural unloading-induced κB target genes. While κB regulates Runx1, Runx1 activity does not seem to involve activation of FoxO or NF-κB-mediated transcription during disuse muscle atrophy [35]. We show that Bcl-3 and p50 have structural gene targets (Table S1) and some of these share the same functional category (e.g., channels, contractile proteins) as targets of Runx1 but none were the same genes as described by Wang et al. [35].

The *Tnfrsf12a* gene was unique among the 14 genes studied for ChIP as it showed an increase in Bcl-3 binding, a moderate increase in p50 binding, and increased p65 binding. The lack of increased p65 binding to putative κB sites in all but one of the 14 genes studied by ChIP is consistent with previous work suggesting a lack of primary involvement of p65 in unloading atrophy [8]. On the other hand, dominant negative overexpression of either IκBα SR, IKKα, or IKKβ inhibited unloading atrophy by ∼50% suggesting that p65 may be involved in regulating some atrophy genes. In the case of *Tnfrsf12a*, regulation via κB proteins may involve a complex of p65:p50:Bcl-3 binding as previously described in response to TNFα [29].

*Tnfrsf12a*, found to be a direct κB target is a receptor for a member of the TNF superfamily also known as TWEAK. This cytokine receptor is of interest as it has recently been shown to be increased with denervation atrophy [36]. In addition, TWEAK is known to induce muscle wasting in whole muscle [37] and it was required for denervation atrophy [36]. As with other pro-inflammatory cytokines, NF-κB is involved in mediating the wasting due to TWEAK in skeletal muscle [37]. We show here that the upregulation of the TWEAK receptor requires κB proteins. Whether unloading also involves TWEAK binding to its receptor is not yet clear.

*IP-10* was also found to be a direct κB target with unloading. It is a chemokine and a known NF-κB target gene in non-muscle cells types [29], [38]. The strong upregulation of this gene together with the robust increase in p50 and Bcl-3 binding to *IP-10* in unloaded muscle suggest there may be some role for immune modulator genes in this type of atrophy. In control muscle there was significant p65 binding to *IP-10* but this was undetectable at 6 days of unloading. In response to TNFα however, p65 binding to bona fide κB sites is increased [29]. The *IP-10* and *Tnfrsf12a* data suggest further study of selected chemokines and cytokines/receptors in unloading atrophy. Consistent with previous work however [7], [8], the present data supports the idea that in the absence of inflammatory response [19], p65 does not appear to have a major role in binding κB sites on atrophy genes, in contrast with its involvement in atrophy when inflammatory triggers are involved [4], [10], [13].

From these studies we have created a list of NF-κB regulated genes in unloading atrophy, and, we determined that at least 7 of the 14 genes studied in detail using ChIP are direct targets of, p50 and Bcl-3. These data in combination with the lack of increases in p65 binding at κB sites in 13 of the 14 upregulated genes studied, again suggests a primary role of p50 and Bcl-3 rather than the canonical NF-κB transcription factor, p65 (RelA). There are very likely other transcription factors required for unloading atrophy; gene regulation is created by interactions of several factors in complexes or in separate sites on the promoter and regulatory regions. However, it is clear that loss of either of these κB proteins in the knockout animals eliminates or profoundly reduces the atrophy produced by unloading and therefore our candidate list not only outlines the scope of genes whose products create muscle wasting, but it gives us the keystone genomic sites of regulation with which we will be able to understand disuse atrophy at the molecular genetic level.

## Materials and Methods

### Mice and Hind limb Unloading

Six week-old male wild type mice (B6129PF2/J) and age/sex matched *Nfkb1^−/−^* mice (B6;129P2-*Nfkb1^tm1Bal^*/J) and *Bcl3^−/−^* mice (FVB; 129P2-Bcl3^tm1Ver^/J) were purchased from the Jackson Laboratory (Bar harbor, ME). The B6129PF2/J strain used for wild type has all of the 129 background which was part of the knockout cloning and insertion (the embryonic stem cell background) for each of these mutants. Animals were provided with chow and water ad libitum and housed individually in Boston University Animal Care Facility. After 3 days of acclimation, mice were randomly assigned to weight-bearing (WB) or hind limb unloaded (HU) groups. Mice in the HU group had their hind limbs elevated off the cage floor for 6 days to induce unloading induced muscle atrophy. The sixth day of unloading is a key point in the atrophy process based on a large literature, and in particular based on a previous time course study we performed on unloaded rat soleus muscle [18].

The HU was performed using the methods that we have previously published [39] with slight modifications for the mice. Briefly, mice were lightly anesthetized with ketamine/xylazine (40mg/kg;5mg/kg, i.p.). A strip of adhesive foam pad (ALIMED Inc, MA) was folded in half and the adhesive surface was loosely applied longitudinally along the proximal 2/3 of the tail. Elastic tape (Elastoplast, Medco Supply Co., NY) was wrapped circumferentially around the adhesive foam. A wire was passed through the folded end of adhesive foam pad and then was drawn up to pulley attached to a 360° swivel hook at the top of the cage; the cage walls were extended in height by 10 inches of Plexiglas on 4 sides to allow room for the vertical suspension. The length of the wire was adjusted so that the toes of the hind limbs touched the cage floor only during full hind limb extension. The use of animals in this study was approved by the Institutional Animal Care and Use Committee of Boston University (protocol number 09-012).

### Muscle harvesting and RNA isolation

Six days after hind limb unloading, mice were anesthetized with ketamine/xylazine (100mg/kg; 13mg/kg). Gastrocnemius and plantaris muscles were harvested from weight bearing and hind limb unloaded wild type mice, *Nfkb1* ^−/−^ mice, and *Bcl3^−/−^* mice and processed immediately for RNA isolation or ChIP assay. Total RNA was extracted using TRIzol reagent (Invitrogen, Carlsbad, CA) according to manufacturer's instructions. Extracted total RNA was treated with RNase-Free DNase I (Qiagen, Valencia, CA) to remove any DNA contamination and purified using an RNeasy Mini kit (Qiagen, Valencia, CA) as previously described [40]. Total RNA was quantified by absorption spectrophotometry at 260 and 280nm and quality of total RNA was monitored on a 1% denaturing agarose gel and by a Bioanalyzer 2100 (Agilent, Palo Alto, CA).

### Microarray processing

For microarray analysis 24 mice were used based on 4 mice per group, 3 mouse stains, and 2 conditions (weight-bearing and unloaded). Total RNA samples (n = 4 per group) were sent to Partners HealthCare Center for Personalized Genetic Medicine for mRNA labeling, hybridization, and scanning using the Affymetrix system (Santa Clara, CA). In brief, double stranded cDNA was synthesized by GeneChip® Two-Cycle cDNA Synthesis Kit (P/N 900432). Biotin-labeled cRNA was then generated and purified by GeneChip IVT labeling kit (P/N 900449) and cRNA Cleanup kit (P/N 900547). 15µg of labeled fragmented cRNA was hybridized on to Affymetrix GeneChip Mouse Genome 430A 2.0 Array, then washed and stained according to manufacturer's protocol. The 430A 2.0 Array measures expression levels of 14,000 characterized genes. The microarray slides were scanned using a GeneChip® Scanner 3000. For data extraction and quality control, Affymetix GeneChip® operation software was used, which created a single intensity value for each probe-set assayed in each sample in a .cel file. Data files were deposited into the MIAME compliant NCBI Gene Expression Omnibus (GEO) (http://www.ncbi.nlm.nih.gov/geo/), accession number GSE23497.

### Data Analysis

Affymetrix .cel files were imported into GenePattern software 3.2.1 (Broad Institute, Cambridge, MA) and a matrix containing an expression value per probe set for each sample was generated by Expression File Creator Module using the Robust Multi-array Average (RMA) algorithm and quantile normalization [41]. Gene probes were filtered according to the following criteria: maximum expression value/minimum expression value ≥1.7, minimum expression variation (maximum value−minimum value) ≥9, expression value greater than 20 or less than 20,000. Genes complying with these criteria were further processed by the ComparativeMarkerSelection Module Version7. By default, ComparativeMarkerSelection compared the mean difference in gene expressions between weight bearing and unloaded muscle samples using two-way parametric t-tests and computed false discovery rate for each probe set using the *Q*-value [42]. *P*-value<0.05 and *q*-value≤0.05 were used to identify genes that were significantly differentially expressed after hind limb unloading.

We also carried out analyses for the wild type vs. knockouts in the weight bearing condition as a control for any change in gene expression that might arise from the knockout itself. We found no significant differences in muscle gene expression in wild type vs. *Bcl3* ^−/−^, but 17 genes in the *Nfkb1^−/−^* case were lower than wild type. Only one of those 17 was in our list of genes that could be a target of p50 and so it was removed from our results.

### Quantitative Real time-PCR (QRT-PCR)

Total RNA from 3 strains of mice (two knockouts and one wild type) for 2 conditions (WB and HU) was isolated as described above with a sample size of 6 per group. 10µg of total RNA was reverse transcribed in a 100µl reaction using a High Capacity cDNA Reverse Transcription Kit (Applied Biosystems, Foster City, CA) according to manufacturer's instructions. Synthesized cDNA (0.9 µl) was then amplified with Taqman Gene Expression Master mix, and gene-specific TaqMan Gene Expression Assay Buffer (Applied Biosystems). The sequence targeted by each TaqMan probe is given in Table S3. Amplification was performed by ABI 7300 Real-Time PCR System at following temperature: 50°C for 2min, 95°C for 10min, followed by 40 cycles at 95°C for 15sec and 60°C for 1min. All samples were run in triplicate and quantified according to the corresponding gene-specific standard curve. For the 14 genes for which we measured mRNA expression, each gene was normalized to GAPDH expression, which was not different due to mouse strain or to hind limb unloading.

### Statistical Analysis

Methods used for statistical analysis of the microarray data have been described above. All other statistical analysis was performed using the SPSS software package for windows version 15.0 (SPSS Inc, Chicago, IL). For qPCR experiments (n = 6 per group), Levene's test was first performed to compare the variance between weight bearing and hind limb unloaded muscles. If 2 groups showed an equal variance, an equal-variance t-test was performed to determine whether gene expression was upregulated after unloading. If 2 groups showed unequal variance, an unequal-variance t-test was performed. *P*-value less than 0.05 was considered statistically significant.

### ChIP Procedure

Isolation of nuclei from muscle and subsequent successful ChIP assay is infrequent in the literature and methods details are scarce. Our method begins with a modification of Andrews et al. [43]. Gastrocnemius plus plantaris muscles from weight bearing and hind limb unloaded mice were removed, weighed, and immersed in 1 ml of the nuclear isolation buffer (NIB: 10mM of HEPES pH7.5, 10mM MgCl_2_, 60mM KCl, 300mM sucrose, 0.1mM EDTA pH8.0, 0.1% Triton X-100, 1mM DTT, 0.15mM spermine and 0.5mM spermidine, 0.1mM PMSF, 2µg/ml aprotinin, 2µg/ml leupeptin). The muscle was finely minced using sterile scalpels and transferred to a 1.5 ml Eppendorf tube. The minced muscle was crosslinked by adding fresh formaldehyde to a final concentration of 1% and rotating at room temperature (RT) for 15 minutes. Crosslinking was stopped by adding glycine to a final concentration of 0.125M and rotating at RT for 5 minutes. Tubes were centrifuged at 1500*×g* for 5 minutes at 4°C and washed twice with PBS containing 1× protease inhibitor cocktail [(PIC), Roche Applied Bioscience]. After the last spin, the supernatant was discarded and the pellet was snap frozen in liquid nitrogen and stored at −80°C. After thawing, each pellet was suspended in 1.5 ml of NIB, transferred to a 14 ml round bottom tube (BD Falcon), and homogenized mechanically. The homogenates were centrifuged at 180*×g* for 1 minute and supernatants discarded. Pellets were re-suspended in 1.5 ml NIB and homogenized again to maximize the number of nuclei released from myofibrillar material. Homogenates from both legs of the same mouse were pooled and passed though a 100 µm cell strainer (BD Falcon) by centrifugation at 1100*×g* for 5 minutes at 4°C to filter residual myofibrillar material from the nuclei. The filter process was repeated with a 40 µm cell strainer (BD Falcon), and then nuclei pelleted by centrifugation at 1100*×g* for 5 minutes. Each cell strainer was then washed twice with 2 ml of ice cold PBS containing 1× PIC, then centrifuged to pellet any nuclei stuck in the strainer.

The nuclei were processed for ChIP using modifications of the method described by Tachibana et al. [44]. Nuclei were re-suspended in sonication buffer with PIC. Aliquots of 350 to 400 µl were sonicated with a Sonic Dismembrator Model 100 (Fisher) at setting 2 for 30 seconds with one minute in between on ice, for 9 to 12 cycles. Nuclear debris was pelleted by spinning the sonicated material at maximum speed in a microfuge for 5 minutes, and the supernatant containing fragmented chromatin was saved for immunoprecipitation. The extent of sonication was evaluated by removing 10 µl supernatant for treatment with Pronase, RNase A, and reverse crosslinked, and then DNA was extracted with phenol/chloroform and precipitated with ethanol. DNA was size fractionated on a 1% agarose gel where approximately 90% of the DNA was visualized as a smear between 100–600 bp. Equal amounts of fragmented chromatin from each group, weight bearing and hind limb unloaded, were diluted in IP dilution buffer [44] and pre-cleared twice using the Protein G conjugated magnetic beads (Dynabeads, Invitrogen) that had been blocked overnight with BSA and sonicated fish sperm DNA (Roche Applied Bioscience). The beads were pelleted by centrifugation and the supernatant containing the chromatin was aliquoted: a 100 µl aliquot as an input sample not immunopreciptated, and four aliquots with equal amount of the remaining supernatant (∼700–1000 µl each). One aliquot was incubated with IgG as a negative control and the other three chromatin aliquots were immunoprecipitated by incubation with 10 µg of antibody (Santa Cruz), directed against either p65 (SC-372X), p50 (SC-1190X), or Bcl3 (SC-185X) for 16 hours at 4°C. The antibody-chromatin complexes were warmed to RT in a water bath and an equal volume of pre-blocked magnetic beads was added and placed on a rotator for 15 minutes at RT. The tubes were then placed in a magnetic tube rack (Magnasphere separation stand, Promega) to allow capture of beads on the side of the tube. The liquid was removed and beads were washed in a succession of buffers according to Tachibana et al. [44]. Finally the conjugates were eluted from the beads for 30 minutes. The protein and RNA were removed from the immunoprecipitated samples and the input sample by incubation with Pronase and RNase A for 2 hrs at 42°C. The sample and input tubes were processed to reverse the crosslinking and DNA was precipitated in ethanol with glycogen as carrier. The DNA from ChIP was re-suspended in 30 µl of Tris-low EDTA buffer (10 mM Tris 8.0, 0.1 mM EDTA). For PCR, 1/15^th^ of the each DNA sample and 1/100^th^ of each input were used as templates in a standard Platinum Taq PCR reaction for 32 cycles. PCR products were separated on a 10% TBE gel (Ready Gel, BioRad) and stained by SYBR Gold.

NF-κB sites for study were selected by the CLOVER algorithm of Motifviz (http://biowulf.bu.edu/MotifViz/), which uses the JASPAR database (http://jaspar.genereg.net/) for its binding matrices. We selected κB sites for ChIP that had the best score for matching the κB consensus sequence using the JASPAR matrix ID: MA0061.1. Conservation between mouse and human gene sequences was determined by locating the κB site in the mouse genome using Blat and displaying the comparative sequences at that site in the University of California Santa Cruz gene browser (http://genome.ucsc.edu/). Primers were designed with Primer 3.0 from the online workpage (http://frodo.wi.mit.edu/primer3/). The size of the PCR products containing the κB sites was between 190 and 400bp for all 14 ChIP assays.

## Supporting Information

Table S1 Muscle weights for weight bearing (WB) and hind limb unloaded (HU) mice.
(DOC)

Table S2 Complete list of atrophy genes that are target genes for p50 or Bcl-3.
(DOC)

Table S3 TaqMan probes used for quantitative real time-PCR.
(DOC)
