# Supplementary material for: Identification of Genes that Elicit Disuse Muscle Atrophy via the Transcription Factors p50 and Bcl-3
Source: PLoS One. 2011 Jan 13;6(1):e16171. doi: 10.1371/journal.pone.0016171 (PMC3020958; doi:10.1371/journal.pone.0016171)
Supplement: Table S1 — Muscle weights for weight bearing (WB) and hind limb unloaded (HU) mice. (DOC) [file pone.0016171.s001.doc]

**Table S1.**  Muscle weights for weight bearing (WB) and hind limb unloaded (HU) mice.

|  | **Muscle mass/ body mass (mg/g)** | |  |
| --- | --- | --- | --- |
| **Mice strain** | **WB** | **HU** | **Fold change** |
| WT | 5.22 ± 0.11 | 4.30 ± 0.16 | 18%* |
| Nfkb1^-/-^ | 5.50 ± 0.04 | 5.13 ± 0.08 | 6.7%* |
| Bcl-3^-/-^ | 4.86 ± 0.07 | 4.55 ± 0.16 | n.s. |

Muscle is gastrocnemius plus plantaris. Values are mean ± SEM. * indicates different from WB value (*P*<0.05). n.s. indicates no statistical difference from WB value.
