# Supplementary material for: Identification of Genes that Elicit Disuse Muscle Atrophy via the Transcription Factors p50 and Bcl-3
Source: PLoS One. 2011 Jan 13;6(1):e16171. doi: 10.1371/journal.pone.0016171 (PMC3020958; doi:10.1371/journal.pone.0016171)
Supplement: Table S2 — Complete list of atrophy genes that are target genes for p50 or Bcl-3. (DOC) [file pone.0016171.s002.doc]

**Table S2.** Complete list of atrophy genes that are target genes for p50 or Bcl-3.

| **Feature** | **Gene Name, Gene ID** | **FC** | **p50 target** | **Bcl-3 target** |
| --- | --- | --- | --- | --- |
| **Anti-oxidant** | | | | |
| 1449106_at | glutathione peroxidase 3, Gpx3 | 1.72 | N | Y |
| 1451503_at | nucleolar protein 3 (apoptosis repressor with CARD domain), Nol3 | 1.65 | Y | Y |
| 1421148_a_at | Tia1 cytotoxic granule-associated RNA binding protein-like 1, Tial1 | 1.51 | Y | Y |
| 1434557_at | huntingtin interacting protein 1, Hip1 | 1.43 | Y | Y |
| **Binding** | | | | |
| 1448760_at | zinc finger protein 68, Zfp68 | 1.58 | Y | Y |
| 1419281_a_at | zinc finger protein 259, Zfp259 | 1.55 | Y | Y |
| 1417321_at | zinc finger, CCHC domain containing 7, Zcchc7 | 1.52 | Y | Y |
| 1426084_a_at | torsin A interacting protein 1, Tor1aip1 | 1.46 | N | Y |
| 1417088_at | zinc finger protein 346, Zfp346 | 1.44 | Y | Y |
| **Cell adhesion** | | | | |
| 1416039_x_at | cysteine rich protein 61, Cyr61 | 2.28 | Y | Y |
| 1453556_x_at | CD99 antigen, Cd99 | 1.65 | Y | Y |
| 1418393_a_at | integrin alpha 7, Itga7 | 1.62 | Y | Y |
| 1421811_at | thrombospondin 1 /// similar to thrombospondin 1, Thbs1 | 1.58 | Y | Y |
| 1419613_at | collagen, type VII, alpha 1, Col7a1 | 1.56 | Y | Y |
| 1460302_at | thrombospondin 1, Thbs1 | 1.46 | N | Y |
| 1436042_at | talin 1, Tln1 | 1.4 | Y | Y |
| **Cell cycle** | | | | |
| 1424105_a_at | pituitary tumor-transforming 1, Pttg1 | 3.14 | Y | Y |
| 1424638_at | cyclin-dependent kinase inhibitor 1A (P21), Cdkn1a | 2.56 | N | Y |
| 1417850_at | retinoblastoma 1, Rb1 | 2.23 | Y | Y |
| 1416028_a_at | hematological and neurological expressed sequence 1, Hn1 | 1.9 | Y | Y |
| 1417516_at | DNA-damage inducible transcript 3, Ddit3 | 1.59 | N | Y |
| 1417619_at | growth arrest and DNA-damage-inducible, gamma interacting protein 1, Gadd45gip1 | 1.59 | Y | Y |
| **Chromatin modification** | | | | |
| 1420477_at | nucleosome assembly protein 1-like 1, Nap1l1 | 2.21 | Y | Y |
| 1424760_a_at | SET and MYND domain containing 2, Smyd2 | 1.95 | N | Y |
| 1429038_at | DET1 and DDB1 associated 1, Dda1 | 1.81 | Y | Y |
| 1426810_at | jumonji domain containing 1A, Jmjd1a | 1.77 | N | Y |
| 1420376_a_at | H3 histone, family 3A /// H3 histone, family 3B, H3f3a /// H3f3b | 1.59 | Y | Y |
| 1451167_at | coiled-coil domain containing 101, Ccdc101 | 1.56 | Y | Y |
| 1437210_a_at | bromodomain containing 2, Brd2 | 1.53 | N | Y |
| 1449080_at | histone deacetylase 2, Hdac2 | 1.5 | Y | Y |
| 1418367_x_at | histone cluster 2, H3c1 | 1.5 | Y | Y |
| 1425591_a_at | chromatin modifying protein 2A, Chmp2a | 1.43 | Y | Y |
| **Cytoskeleton** | | | | |
| 1455493_at | synaptic nuclear envelope 1, Syne1 | 1.8 | N | Y |
| 1448665_at | dystrophin, muscular dystrophy, Dmd | 1.7 | Y | Y |
| 1451200_at | kinesin family member 1B, Kif1b | 1.57 | Y | Y |
| 1434937_at | MYC binding protein 2, Mycbp2 | 1.55 | N | Y |
| 1439463_x_at | high mobility group box 1 | 1.55 | Y | Y |
| 1450650_at | myosin X, Myo10 | 1.51 | Y | Y |
| 1416499_a_at | dynactin 6, Dctn6 | 1.5 | Y | Y |
| 1418986_a_at | ubiquitously expressed transcript, Uxt | 1.47 | Y | Y |
| 1417626_at | phosphodiesterase 4D interacting protein (myomegalin), Pde4dip | 1.44 | Y | Y |
| 1416179_a_at | radixin, Rdx | 1.43 | Y | Y |
| 1428835_at | myosin, heavy polypeptide 14, Myh14 | 1.43 | Y | Y |
| **Development** | | | | |
| 1427201_at | musculoskeletal, embryonic nuclear protein 1, Mustn1 | 3.5 | N | Y |
| 1416067_at | interferon-related developmental regulator 1, Ifrd1 | 2.7 | Y | Y |
| 1420895_at | transforming growth factor, beta receptor I, Tgfbr1 | 1.75 | N | Y |
| 1423606_at | periostin, osteoblast specific factor, Postn | 1.72 | N | Y |
| 1428853_at | patched homolog 1, Ptch1 | 1.63 | Y | Y |
| 1431475_a_at | homeo box A10, Hoxa10 | 1.45 | Y | Y |
| 1448266_at | endothelial differentiation-related factor 1, Edf1 | 1.4 | Y | Y |
| **ECM** | | | | |
| 1426808_at | lectin, galactose binding, soluble 3, Lgals3 | 5.33 | N | Y |
| **Immune** | | | | |
| 1428346_at | TRAF type zinc finger domain containing 1, Trafd1 | 1.55 | Y | Y |
| 1453256_at | polymerase (RNA) III (DNA directed) polypeptide C, Polr3c | 1.42 | Y | Y |
| **Metabolism** | | | | |
| 1449526_a_at | glycerophosphodiester phosphodiesterase domain containing 3, Gdpd3 | 9.85 | Y | Y |
| 1451290_at | microtubule-associated protein 1 light chain 3 alpha, Map1lc3a | 2.06 | N | Y |
| 1434442_at | starch binding domain 1, Stbd1 | 1.9 | Y | Y |
| 1450660_at | 6-pyruvoyl-tetrahydropterin synthase, Pts | 1.86 | Y | Y |
| 1448111_at | cytidine 5'-triphosphate synthase 2, Ctps2 | 1.85 | Y | Y |
| 1434542_at | glutamic pyruvate transaminase (alanine aminotransferase) 2, Gpt2 | 1.8 | N | Y |
| 1456251_x_at | translocator protein, Tspo | 1.74 | Y | Y |
| 1451257_at | acyl-CoA synthetase long-chain family member 6, Acsl6 | 1.73 | N | Y |
| 1448736_a_at | hypoxanthine guanine phosphoribosyl transferase 1, Hprt1 | 1.64 | Y | Y |
| 1416432_at | 6-phosphofructo-2-kinase/fructose-2,6-biphosphatase 3, Pfkfb3 | 1.62 | N | Y |
| 1422902_s_at | meningioma expressed antigen 5 (hyaluronidase), Mgea5 | 1.61 | N | Y |
| 1449968_s_at | acyl-CoA thioesterase 9, Acot9 /// acyl-CoA thioesterase 10, Acot10 | 1.61 | Y | Y |
| 1416352_s_at | glycoprotein, synaptic 2, Gpsn2 | 1.57 | Y | Y |
| 1425764_a_at | branched chain aminotransferase 2, mitochondrial, Bcat2 | 1.57 | Y | Y |
| 1415965_at | stearoyl-Coenzyme A desaturase 1, Scd1 | 1.56 | Y | Y |
| 1423215_at | signal peptidase complex subunit 2 homolog (S. cerevisiae), Spcs2 | 1.52 | Y | Y |
| 1448143_at | aldehyde dehydrogenase 2, mitochondrial, Aldh2 | 1.5 | Y | Y |
| 1425515_at | phosphatidylinositol 3-kinase, regulatory subunit, polypeptide 1 (p85 alpha), Pik3r1 | 1.48 | N | Y |
| 1450966_at | carnitine O-octanoyltransferase, Crot | 1.45 | Y | Y |
| 1452781_a_at | Mpv17 transgene, kidney disease mutant /// general transcription factor IIIC, polypeptide 2, beta, Gtf3c2 /// Mpv17 | 1.43 | Y | Y |
| 1422608_at | cAMP-regulated phosphoprotein 19, Arpp19 | 1.42 | Y | Y |
| 1420502_at | spermidine/spermine N1-acetyl transferase 1, Sat1 | 1.42 | Y | Y |
| 1437711_x_at | ornithine decarboxylase, structural 1, Odc1 | 1.4 | Y | Y |
| 1448495_at | tissue specific transplantation antigen P35B, Tsta3 | 1.4 | Y | Y |
| **Others** | | | | |
| 1448199_at | ankyrin repeat domain 10, Ankrd10 | 2.4 | N | Y |
| 1426464_at | nuclear receptor subfamily 1, group D, member 1, Nr1d1 | 1.85 | Y | Y |
| 1433509_s_at | receptor accessory protein 1, Reep1 | 1.79 | Y | Y |
| 1439405_x_at | Cyclin N-terminal domain containing 1, Cntd1 | 1.54 | Y | Y |
| 1433674_a_at | small nucleolar RNA host gene (non-protein coding) 1, Snhg1 | 1.52 | Y | Y |
| 1428389_s_at | WD repeat domain 43, Wdr43 | 1.46 | Y | Y |
| 1424759_at | arrestin domain containing 4, Arrdc4 | 1.45 | Y | Y |
| 1451442_at | coiled-coil domain containing 104, Ccdc104 | 1.44 | Y | Y |
| **Muscle contraction** | | | | |
| 1422580_at | myosin, light polypeptide 4, Myl4 | 1.93 | Y | Y |
| 1450813_a_at | troponin I, skeletal, slow 1, Tnni1 | 1.47 | Y | Y |
| 1456623_at | tropomyosin 1, alpha, Tpm1 | 1.41 | Y | Y |
| **Proliferation** | | | | |
| 1426083_a_at | B-cell translocation gene 1, anti-proliferative /// similar to myocardial vascular inhibition factor, Btg1 | 1.67 | Y | Y |
| 1460542_s_at | transformed mouse 3T3 cell double minute 4, Mdm4 | 1.64 | Y | Y |
| **Protein degradation** | | | | |
| 1417522_at | F-box protein 32, Fbxo32 | 2.85 | N | Y |
| 1422650_a_at | RIO kinase 3 (yeast), Riok3 | 1.94 | N | Y |
| 1417480_at | f-box protein 9, Fbxo9 | 1.8 | N | Y |
| 1451310_a_at | cathepsin L, Ctsl | 1.61 | N | Y |
| 1448824_at | ubiquitin-conjugating enzyme E2, J1, Ube2j1 | 1.61 | Y | Y |
| 1453623_a_at | RAD23a homolog (S. cerevisiae), Rad23a | 1.53 | Y | Y |
| 1416290_a_at | proteasome (prosome, macropain) 26S subunit, ATPase, 4, Psmc4 | 1.53 | Y | Y |
| 1422459_a_at | proteasome (prosome, macropain) 26S subunit, non-ATPase, 13, Psmd13 | 1.51 | Y | Y |
| 1423568_at | proteasome (prosome, macropain) subunit, alpha type 7, Psma7 | 1.51 | Y | Y |
| 1460698_a_at | SEC11 homolog C (S. cerevisiae), Sec11c | 1.51 | Y | Y |
| 1423269_a_at | neural precursor cell expressed, developmentally down-regulated gene 4-like, Nedd4l | 1.47 | Y | Y |
| 1451971_at | cullin 4A, Cul4a | 1.47 | Y | Y |
| 1415831_at | proteasome (prosome, macropain) 26S subunit, non-ATPase, 2, Psmd2 | 1.47 | Y | Y |
| 1426400_a_at | calpain, small subunit 1, Capns1 | 1.46 | Y | Y |
| 1448591_at | cathepsin S, Ctss | 1.46 | Y | Y |
| 1417052_at | proteasome (prosome, macropain) subunit, beta type 3, Psmb3 | 1.45 | Y | Y |
| 1435431_at | proteasome (prosome, macropain) assembly chaperone 4, Psmg4 | 1.44 | Y | Y |
| 1438984_x_at | proteasome (prosome, macropain) subunit, beta type 4, Psmb4 | 1.44 | Y | Y |
| 1448883_at | legumain, Lgmn | 1.41 | Y | Y |
| 1425859_a_at | proteasome (prosome, macropain) 26S subunit, non-ATPase, 4, Psmd4 | 1.4 | Y | Y |
| 1418020_s_at | carboxypeptidase D, Cpd | 1.4 | Y | Y |
| **RNA processing** | | | | |
| 1422885_at | small nuclear ribonucleoprotein D3, Snrpd3 | 1.89 | N | Y |
| 1454793_x_at | DEAD (Asp-Glu-Ala-Asp) box polypeptide 5, Ddx5 | 1.75 | Y | Y |
| 1436494_x_at | TRM1 tRNA methyltransferase 1 homolog (S. cerevisiae), Trmt1 | 1.66 | Y | Y |
| 1422849_a_at | poly(A) binding protein, nuclear 1, Pabpn1 | 1.64 | Y | Y |
| 1442744_at | RNA binding motif protein 39, Rbm39 | 1.54 | Y | Y |
| 1426793_a_at | ribosomal protein L14 /// similar to Ribosomal protein L14, Rpl14 | 1.52 | Y | Y |
| 1434390_at | Heterogeneous nuclear ribonucleoprotein U, Hnrnpu | 1.52 | N | Y |
| 1423130_a_at | splicing factor, arginine/serine-rich 5 (SRp40, HRS), Sfrs5 | 1.48 | Y | Y |
| 1421063_s_at | small nuclear ribonucleoprotein N, Snrpn | 1.46 | Y | Y |
| 1455696_a_at | PRP4 pre-mRNA processing factor 4 homolog B (yeast), Prpf4b | 1.43 | N | Y |
| 1454689_at | serine/arginine repetitive matrix 1, Srrm1 | 1.42 | Y | Y |
| 1415807_s_at | splicing factor, arginine/serine-rich 2 (SC-35), Sfrs2 | 1.41 | Y | Y |
| **Signaling** | | | | |
| 1424831_at | copine II, Cpne2 | 4.94 | Y | Y |
| 1418572_x_at | tumor necrosis factor receptor superfamily, member 12a, Tnfrsf12a | 2.68 | Y | Y |
| 1428057_a_at | AHNAK nucleoprotein (desmoyokin), Ahnak | 2.41 | Y | Y |
| 1416029_at | Kruppel-like factor 10, Klf10 | 2.08 | N | Y |
| 1425241_a_at | WD repeat and SOCS box-containing 1, Wsb1 | 1.83 | Y | Y |
| 1424613_at | G protein-coupled receptor, family C, group 5, member B, Gprc5b | 1.79 | Y | Y |
| 1427005_at | polo-like kinase 2 (Drosophila), Plk2 | 1.79 | Y | Y |
| 1424542_at | S100 calcium binding protein A4, S100a4 | 1.76 | Y | Y |
| 1436566_at | Rab40b, member RAS oncogene family, Rab40b | 1.74 | Y | Y |
| 1426465_at | discs, large homolog-associated protein 4 (Drosophila), Dlgap4 | 1.74 | Y | Y |
| 1415712_at | zinc finger, RAN-binding domain containing 1, Zranb1 | 1.73 | Y | Y |
| 1424176_a_at | annexin A4, Anxa4 | 1.72 | Y | Y |
| 1427006_at | Rap guanine nucleotide exchange factor (GEF) 1, Rapgef1 | 1.69 | Y | Y |
| 1426602_at | v-raf murine sarcoma 3611 viral oncogene homolog, Araf | 1.64 | Y | Y |
| 1454060_a_at | neuroblastoma ras oncogene, Nras | 1.62 | Y | Y |
| 1422886_a_at | CDC like kinase 4, Clk4 | 1.62 | N | Y |
| 1439962_at | RIKEN cDNA 2310010J17 gene, 2310010J17Rik | 1.61 | Y | Y |
| 1430295_at | guanine nucleotide binding protein, alpha 13, Gna13 | 1.61 | Y | Y |
| 1448582_at | catenin, beta like 1, Ctnnbl1 | 1.59 | Y | Y |
| 1421321_a_at | neuroepithelial cell transforming gene 1, Net1 | 1.58 | Y | Y |
| 1428011_a_at | Erbb2 interacting protein, Erbb2ip | 1.57 | Y | Y |
| 1418822_a_at | ADP-ribosylation factor 6, Arf6 | 1.57 | Y | Y |
| 1431428_a_at | nitric oxide synthase interacting protein, Nosip | 1.56 | Y | Y |
| 1451063_at | syntaxin binding protein 4, Stxbp4 | 1.56 | Y | Y |
| 1421324_a_at | thymoma viral proto-oncogene 2 /// similar to serine/threonine kinase, Akt2 /// LOC100048123 | 1.55 | Y | Y |
| 1434820_s_at | protein kinase inhibitor, gamma, Pkig | 1.54 | Y | Y |
| 1424893_at | nuclear distribution gene E-like homolog 1 (A. nidulans), Ndel1 | 1.53 | Y | Y |
| 1426044_a_at | protein kinase C, theta, Prkcq | 1.48 | Y | Y |
| 1425202_a_at | ankyrin 3, epithelial, Ank3 | 1.46 | N | Y |
| 1448676_at | calcium/calmodulin-dependent protein kinase II, beta, Camk2b | 1.45 | Y | Y |
| 1417398_at | related RAS viral (r-ras) oncogene homolog 2, Rras2 | 1.44 | Y | Y |
| **Stress** | | | | |
| 1449519_at | growth arrest and DNA-damage-inducible 45 alpha, Gadd45a | 6.08 | N | Y |
| 1416411_at | glutathione S-transferase, mu 2, Gstm2 | 2.74 | N | Y |
| 1422557_s_at | metallothionein 1, Mt1 | 1.91 | Y | Y |
| **Structure** | | | | |
| 1449533_at | transmembrane protein 100, Tmem100 | 5.46 | N | Y |
| 1435526_at | torsin A interacting protein 2, Tor1aip2 | 1.54 | Y | Y |
| 1430421_a_at | transmembrane protein 205, Tmem205 | 1.5 | Y | Y |
| 1434503_s_at | lysosomal-associated membrane protein 2, Lamp2 | 1.5 | Y | Y |
| 1426628_at | transmembrane protein 34, Tmem34 | 1.48 | Y | Y |
| 1455642_a_at | tetraspanin 17, Tspan17 | 1.43 | Y | Y |
| 1424354_at | transmembrane protein 140, Tmem140 | 1.42 | N | Y |
| **Transcription** | | | | |
| 1420991_at | ankyrin repeat domain 1 (cardiac muscle), Ankrd1 | 7.93 | N | Y |
| 1422864_at | runt related transcription factor 1, runx1 | 5.26 | Y | Y |
| 1435458_at | proviral integration site 1, Pim1 | 2.62 | N | Y |
| 1426690_a_at | sterol regulatory element binding transcription factor 1, Srebf1 | 2.42 | Y | Y |
| 1449363_at | activating transcription factor 3, Atf3 | 2.24 | Y | Y |
| 1448293_at | early B-cell factor 1, Ebf1 | 1.71 | Y | Y |
| 1417409_at | Jun oncogene, Jun | 1.65 | Y | Y |
| 1449029_at | MAP kinase-interacting serine/threonine kinase 2, Mknk2 | 1.65 | Y | Y |
| 1428467_at | TAR DNA binding protein, Tardbp | 1.64 | N | Y |
| 1434037_s_at | p300/CBP-associated factor, Pcaf | 1.63 | Y | Y |
| 1449800_x_at | PHD finger protein 7, Phf7 | 1.6 | Y | Y |
| 1426383_at | cryptochrome 2 (photolyase-like) /// similar to mKIAA0658 protein, Cry2 /// LOC100048334 | 1.54 | Y | Y |
| 1455039_a_at | transcriptional regulator, SIN3B (yeast), Sin3b | 1.51 | Y | Y |
| 1451285_at | fusion, derived from t(12;16) malignant liposarcoma (human), Fus | 1.5 | Y | Y |
| 1427162_a_at | ELK4, member of ETS oncogene family, Elk4 | 1.48 | Y | Y |
| 1454149_a_at | cyclin L2, Ccnl2 | 1.48 | Y | Y |
| 1423839_a_at | basic transcription factor 3, Btf3 | 1.45 | Y | Y |
| 1416630_at | inhibitor of DNA binding 3, Id3 | 1.44 | Y | Y |
| 1420870_at | myeloid/lymphoid or mixed-lineage leukemia (trithorax homolog, Drosophila); translocated to, 10, Mllt10 | 1.43 | N | Y |
| 1415997_at | thioredoxin interacting protein, Txnip | 1.43 | N | Y |
| 1422554_at | necdin-like 2, Ndnl2 | 1.41 | Y | Y |
| **Translation** | | | | |
| 1416332_at | cold inducible RNA binding protein, Cirbp | 2.71 | Y | Y |
| 1422660_at | RNA binding motif protein 3, Rbm3 | 2.6 | N | Y |
| 1448325_at | myeloid differentiation primary response gene 116, Myd116 | 2.57 | N | Y |
| 1434004_at | deoxyhypusine synthase, Dhps | 1.78 | Y | Y |
| 1423142_a_at | GTP binding protein 4, Gtpbp4 | 1.77 | Y | Y |
| 1439415_x_at | ribosomal protein S21, Rps21 | 1.63 | Y | Y |
| 1434976_x_at | eukaryotic translation initiation factor 4E binding protein 1, Eif4ebp1 | 1.61 | Y | Y |
| 1423254_x_at | ribosomal protein S27-like, Rps27l | 1.57 | Y | Y |
| 1433631_at | eukaryotic translation initiation factor 5, Eif5 | 1.52 | N | Y |
| 1449323_a_at | ribosomal protein L3, Rpl3 | 1.51 | Y | Y |
| 1460171_at | COP9 (constitutive photomorphogenic) homolog, subunit 5 (Arabidopsis thaliana), Cops5 | 1.5 | Y | Y |
| 1454664_a_at | eukaryotic translation initiation factor 5, Eif5 | 1.49 | Y | Y |
| 1435712_a_at | ribosomal protein S18, Rps18 | 1.48 | Y | Y |
| 1453467_s_at | ribosomal protein S15a, Rps15a | 1.47 | Y | Y |
| 1448472_at | valyl-tRNA synthetase, Vars | 1.47 | Y | Y |
| 1456083_x_at | eukaryotic translation initiation factor 3, subunit C, Eif3c | 1.46 | Y | Y |
| 1450506_a_at | interferon stimulated exonuclease gene 20-like 1, Isg20l1 | 1.46 | Y | Y |
| 1438507_x_at | ribosomal protein L14, Rpl14 | 1.45 | Y | Y |
| 1455141_at | trinucleotide repeat containing 6a, Tnrc6a | 1.44 | Y | Y |
| 1418568_x_at | signal recognition particle 14, Srp14 | 1.43 | Y | Y |
| 1429453_a_at | mitochondrial ribosomal protein L55, Mrpl55 | 1.41 | Y | Y |
| 1448217_a_at | ribosomal protein L27, Rpl27 | 1.4 | Y | Y |
| **Transport** | | | | |
| 1420884_at | sarcolipin, Sln | 20.89 | N | Y |
| 1418852_at | cholinergic receptor, nicotinic, alpha polypeptide 1 (muscle) /// similar to acetylcholine receptor alpha-subunit, Chrna1 | 3.32 | N | Y |
| 1421840_at | ATP-binding cassette, sub-family A (ABC1), member 1, Abca1 | 2.83 | N | Y |
| 1420682_at | cholinergic receptor, nicotinic, beta polypeptide 1 (muscle), Chrnb1 | 2.46 | N | Y |
| 1416371_at | apolipoprotein D, Apod | 2.39 | N | Y |
| 1434513_at | ATPase type 13A3, Atp13a3 | 2.11 | Y | Y |
| 1448568_a_at | solute carrier family 20, member 1, Slc20a1 | 1.7 | Y | Y |
| 1449099_at | LPS-responsive beige-like anchor, Lrba | 1.69 | N | Y |
| 1451665_a_at | adaptor-related protein complex AP-4, sigma 1, Ap4s1 | 1.67 | Y | Y |
| 1451130_at | unconventional SNARE in the ER 1 homolog (S. cerevisiae), Use1 | 1.63 | Y | Y |
| 1422813_at | calcium channel, voltage-dependent, gamma subunit 1, Cacng1 | 1.62 | Y | Y |
| 1416138_at | annexin A7, Anxa7 | 1.62 | Y | Y |
| 1423173_at | N-ethylmaleimide sensitive fusion protein attachment protein beta, Napb | 1.57 | N | Y |
| 1423082_at | Der1-like domain family, member 1, Derl1 | 1.56 | Y | Y |
| 1426344_at | GLE1 RNA export mediator (yeast), Gle1 | 1.5 | Y | Y |
| 1438559_x_at | solute carrier family 44, member 2, Slc44a2 | 1.48 | Y | Y |
| 1434773_a_at | solute carrier family 2 (facilitated glucose transporter), member 1, Slc2a1 | 1.42 | Y | Y |
| 1453063_at | clathrin, light polypeptide (Lcb), Cltb | 1.41 | Y | Y |
| 1418447_at | golgi autoantigen, golgin subfamily a, 5, Golga5 | 1.4 | Y | Y |
| **Non-annotated genes or RIKEN cDNA** | | | | |
| 1450013_at | RIKEN cDNA 2900073G15 gene, 2900073G15Rik | 2.49 | Y | Y |
| 1420820_at | RIKEN cDNA 2900073G15 gene, 2900073G15Rik | 2.07 | Y | Y |
| 1449629_s_at | Transcribed locus, --- | 1.81 | N | Y |
| 1417348_at | RIKEN cDNA 2310039H08 gene, 2310039H08Rik | 1.71 | Y | Y |
| 1433924_at | Transcribed locus, --- | 1.63 | N | Y |
| 1435690_at | RIKEN cDNA 2310008H09 gene, 2310008H09Rik | 1.62 | Y | Y |
| 1434025_at | Transcribed locus, --- | 1.5 | N | Y |
| 1436092_at | Transcribed locus, --- | 1.48 | Y | Y |
| 1435524_at | CDNA clone IMAGE:1196043, --- | 1.48 | N | Y |
| 1428317_at |  | 1.47 | Y | Y |
| 1423947_at | RIKEN cDNA 1110008P14 gene, 1110008P14Rik | 1.45 | Y | Y |
| 1431712_a_at | RIKEN cDNA 2310022A10 gene, 2310022A10Rik | 1.45 | Y | Y |
| 1428529_at | RIKEN cDNA 2810026P18 gene, 2810026P18Rik | 1.44 | N | Y |
| 1428515_at | RIKEN cDNA 2410012H22 gene, 2410012H22Rik | 1.41 | Y | Y |

FC indicates the fold change of gene expression due to hind limb unloading in wild type animals from the microarray analysis. Y= a target gene for p50 or Bcl-3, N= not a target gene for p50 or Bcl-3.
