# Supplementary material for: Identification of Genes that Elicit Disuse Muscle Atrophy via the Transcription Factors p50 and Bcl-3
Source: PLoS One. 2011 Jan 13;6(1):e16171. doi: 10.1371/journal.pone.0016171 (PMC3020958; doi:10.1371/journal.pone.0016171)
Supplement: Table S3 — TaqMan probes used for quantitative real time-PCR. (DOC) [file pone.0016171.s003.doc]

**Table S3.** TaqMan probes used for quantitative real time-PCR.

| **Gene Symbol** | **Gene Name** | **Assay ID** | **Probe Sequence** |
| --- | --- | --- | --- |
| Trim63 | tripartite motif-containing 63 | Mm01185222_m1 | GGAGTGCTCCAGTCGGCCCCTGCAG |
| Fbxo32 | F-box protein 32 | Mm00499523_m1 | TCCTGGAAGGGCACTGACCATCCGT |
| Ubc | ubiquitin C | Mm01198158_m1 | ACAACTCCGTGAGAGAGACGATGCA |
| Fbxo9 | f-box protein 9 | Mm01149125_m1 | CGTTGGGAGCGGCTACATCGAAGAG |
| Psma6 | proteasome (prosome, macropain) subunit, alpha type 6 | Mm00478827_m1 | ACATTTGAACAGACAGTGGAAACTG |
| Psmc4 | proteasome (prosome, macropain) 26S subunit, ATPase, 4 | Mm00821599_g1 | CATCTGTCAGGAGAGTGGAATGTTG |
| Psmg4 | proteasome (prosome, macropain) assembly chaperone 4 | Mm01354373_m1 | ATGTGCAGCCGCTACGACCCCATCC |
| Ctsl | cathepsin L | Mm00515597_m1 | GGCTTGTCAAGAACAGCTGGGGAAG |
| Foxo3 | forkhead box O3 | Mm00490673_m1 | GAAGGGAAGGAGCCGAGCTGGAGCT |
| Runx1 | runt related transcription factor 1 | Mm01213405_m1 | TCGACTCTCAACGGCTCCGGACCTG |
| Ankrd1 | ankyrin repeat domain 1 (cardiac muscle) | Mm00496512_m1 | CAAGGTCAAGAACTGTGCTGGGAAG |
| Tnfrsf12a | tumor necrosis factor receptor superfamily, member 12a | Mm00489103_m1 | GCACCAGGCACCTCCCCATGCTCTA |
| Eif4ebp1 | eukaryotic translation initiation factor 4E binding protein 1 | Mm01962435_g1 | TCTCTGTGTGTGCTGCTGTGGGGGG |
| Cxcl10 | chemokine (C-X-C motif) ligand 10 | Mm99999072_m1 | AGTGGGACTCAAGGGATCCCTCTCG |
| GAPDH | glyceraldehyde-3-phosphate dehydrogenase | Mm03302249_g1 | ACAATGAATACGGCTACAGCAACAG |
